# Crystallographic and Molecular Dynamics Analysis of Loop Motions Unmasking the Peptidoglycan-Binding Site in Stator Protein MotB of Flagellar Motor

**DOI:** 10.1371/journal.pone.0018981

**Published:** 2011-04-20

**Authors:** Cyril F. Reboul, Daniel A. Andrews, Musammat F. Nahar, Ashley M. Buckle, Anna Roujeinikova

**Affiliations:** Department of Microbiology and Department of Biochemistry and Molecular Biology, Monash University, Clayton, Victoria, Australia; Massachusetts Institute of Technology, United States of America

## Abstract

**Background:**

The C-terminal domain of MotB (MotB-C) shows high sequence similarity to outer membrane protein A and related peptidoglycan (PG)-binding proteins. It is believed to anchor the power-generating MotA/MotB stator unit of the bacterial flagellar motor to the peptidoglycan layer of the cell wall. We previously reported the first crystal structure of this domain and made a puzzling observation that all conserved residues that are thought to be essential for PG recognition are buried and inaccessible in the crystal structure. In this study, we tested a hypothesis that peptidoglycan binding is preceded by, or accompanied by, some structural reorganization that exposes the key conserved residues.

**Methodology/Principal Findings:**

We determined the structure of a new crystalline form (Form B) of *Helicobacter pylori* MotB-C. Comparisons with the existing Form A revealed conformational variations in the petal-like loops around the carbohydrate binding site near one end of the β-sheet. These variations are thought to reflect natural flexibility at this site required for insertion into the peptidoglycan mesh. In order to understand the nature of this flexibility we have performed molecular dynamics simulations of the MotB-C dimer. The results are consistent with the crystallographic data and provide evidence that the three loops move in a concerted fashion, exposing conserved MotB residues that have previously been implicated in binding of the peptide moiety of peptidoglycan.

**Conclusion/Significance:**

Our structural analysis provides a new insight into the mechanism by which MotB inserts into the peptidoglycan mesh, thus anchoring the power-generating complex to the cell wall.

## Introduction

The motility protein B (MotB) is a key component of the bacterial flagellar motor. It anchors the MotA/MotB stator ring of the motor to peptidoglycan (PG) of the cell wall and forms part of the proton-conducting channel that couples proton flow to generation of the turning force *via* an as yet unknown mechanism [1], [2]. MotA/MotB units are pre-assembled in the membrane in inactive (closed-channel) form and continuously exchange with the units that form the stator ring [3], [4]. Interaction of the MotA/MotB complex with the flagellar basal body is thought to induce two molecular events: opening of the channel and insertion of the anchor domain of MotB into the PG mesh.

The PG-binding site of MotB resides on the periplasmic C-terminal domain (MotB-C) which shows sequence similarity to outer membrane protein A (OmpA) and related PG-binding proteins [5]. The PG-binding domains are believed to have been acquired by MotBs and other OmpA-like proteins from a common ancestor early in evolution, before MotBs and the outer membrane protein family diverged from each other. The crystal structures of MotB-C from *Helicobacter pylori* [6] and *Salmonella typhimurium* [7] revealed that these proteins share a common OmpA-like fold comprising a mixed four-stranded beta-sheet (order 1423), in which three parallel (β1, β2, β3) and one antiparallel (β4) strands are flanked by alpha helices on one side. Although there is no detailed experimental picture of the association between OmpA-like proteins and PG mesh, previous crystallographic and NMR studies on the proteins of this family established the presence of separate recognition sites for glycan and peptide moieties of PG [6], [8], [9]. Both are located within the three petal-like loops (β1α1 (residues 126–133), β2α2 (163–174) and β3β4 (207–225) in *H. pylori* MotB) at one end of the β-sheet. Although the PG-binding grooves formed by these loops are topologically very similar in different OmpA-like proteins, all five conserved residues (Gly161, Asp164, Leu179, Arg183, Arg226) that are thought to be essential for PG recognition are buried and inaccessible [6]. This suggests that PG binding is preceded by, or accompanied by, some structural reorganization that exposes the key conserved residues.

Here, we report a new crystal form of *H. pylori* MotB-C and present the analysis of its structure and dynamics with a combination of experimental (crystallography) and computational (molecular dynamics simulations) methods. Comparison of the refined structure of this form with the previously reported MotB-C structure [6] provides the evidence for the flexibility of the loops β1α1, β2α2 and β3β4 at the putative PG-binding surface. Principal component analysis (PCA) identifies the concerted opening/closing motions of these loops that are thought to facilitate recognition of MotB by PG by exposing conserved residues in a binding pocket for the peptide moiety of PG.

## Results and Discussion

### Overall Structure

**Figure 1:**
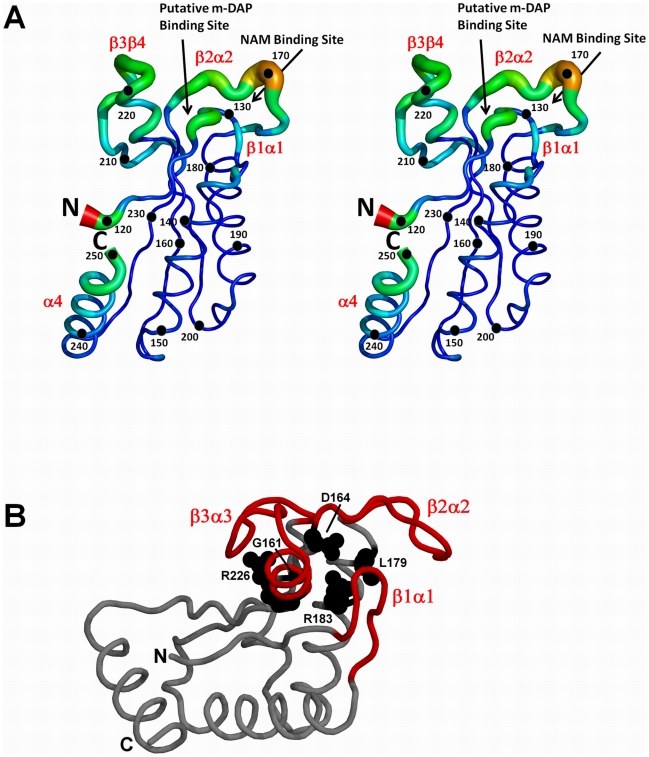
The crystal structure of *H. pylori* MotB-C and the locations of the conserved residues. A: Stereo diagram of the structure of the *H. pylori* MotB-C monomer. The backbone radius is proportional to the average Cα atom RMSD to the mean structure for the superimposition of the total of 16 monomers in the asymmetric units of Form A and Form B crystals. RMSD values were calculated using Theseus [11] and the figure was prepared using PYMOL [12]. The color gradient runs from blue (the smallest RMSD) to red (the largest RMSD). B: The location of the five residues conserved in the family of OmpA-like PG-binding proteins. The MotB-C monomer is drawn using a ribbon representation. Loops β1α1, β2α2 and β3β4, masking these residues, are colored red.

The structure of previously unobserved crystal form (Form B) of recombinant *H. pylori* MotB-C was solved by a molecular replacement approach using AMORE [10]. The coordinates of the previously reported MotB-C structure in a different crystal form (PDB accession code 3CYP [6], hereafter referred to as Form A) were used as a search model. The asymmetric unit of the form B crystal contains 12 subunits. The overall fold of each monomer is very similar to the Form A structure. It contains a mixed four-stranded β-sheet, with three α-helices packing against one face of it, and the fourth forming an N-terminal extension of one of the β-strands (Fig. 1(A)). The loops connecting β-strands with α-helices are short at one end of the β-sheet (bottom side of the molecule shown in Fig. 1(A)) and long at the other. The three longer petal-like loops (β1α1 (residues 126–133), β2α2 (163–174) and β3β4 (207–225)) have been previously implicated in the PG binding by MotB and PG-associated lipoprotein (PAL) [6], [9]. PG-binding domains of MotB and PAL share a significant degree of structural similarity [6] and can be functionally interchanged [13] with only partial loss of activity. This strongly suggests that MotB and PAL share a common molecular mechanism of PG recognition. Previous structural studies on MotB and PAL established that loop β2α2 accommodates the binding site for the N-acetylmuramic acid (NAM) moiety of PG [5], whereas the grove between loops β1α1 and β2α2 binds its peptide moiety containing meso-diaminopimelate (*m*-DAP) [9] (Fig. 1(A)).

### Conserved PG-binding residues are buried

Alignment of aminoacid sequences of the C-terminal domains of MotBs from different bacteria identifies eight conserved residues (Fig. S1). Five of them (Gly161, Asp164, Leu179, Arg183, Arg226) are conserved in the entire OmpA family of PG-binding proteins [6] and are therefore likely to be involved in direct binding to PG or in maintaining the fold around residues recognized by PG. Two residues in particular, Asp164 and Leu179, play a critical role in recognition of the peptide moity of PG. A previous NMR study on the complex between *Haemophilus influenzae* PAL and a synthetic PG precursor [9] demonstrated that the PAL residues Asp71 and Leu82, equivalent to Asp164 and Leu179 in *H. pylori* MotB, form contacts with the *m*-DAP residue of a synthetic PG precursor, involving a hydrogen bond to the side chain of Asp71 and a hydrophobic interaction with the side chain of Leu82. Structural analysis and calculations of the accessible side-chain surface area in Form A and Form B crystals of *H. pylori* MotB-C and in the crystal structure of the periplasmic domain of *Salmonella* MotB [7] (Table 1) show that all five conserved residues are clustered on one side of the molecule and are completely shielded from the solvent by loops (β1α1), (β2α2) and (β3β4) (Fig. 1(B)). Therefore, we hypothesize that PG binding is preceded by or accompanied by some conformational transition in MotB that exposes the key conserved residues.

**Table 1:** aFractional side-chain accessible surface area (asa) (fractional) of MotB residues that are conserved in OmpA-like PG-binding proteins.

Gly161/Gly195	0.00/0.00
Asp164/Asp198	0.01/0.04
Leu179/Leu214	0.00/0.00
Arg183/Arg218	0.00/0.02
Arg226/Arg260	0.01/0.03

Residue numbering is as in *H. pylori* MotB-C/periplasmic domain of *Salmonella* MotB.

a Asa values for *H. pylori* MotB-C were averaged over all subunits in the asymmetric units of Form A and Form B crystals. The average values for the *Salmonella* MotB domain were calculated using the coordinates for the high-resolution crystal form (Research Collaboration for Structural Bioinformatics (RCSB) Protein Data Bank code 2zvy [7]).

### Conformational variability of the conserved loops at the PG binding site

**Figure 2:**
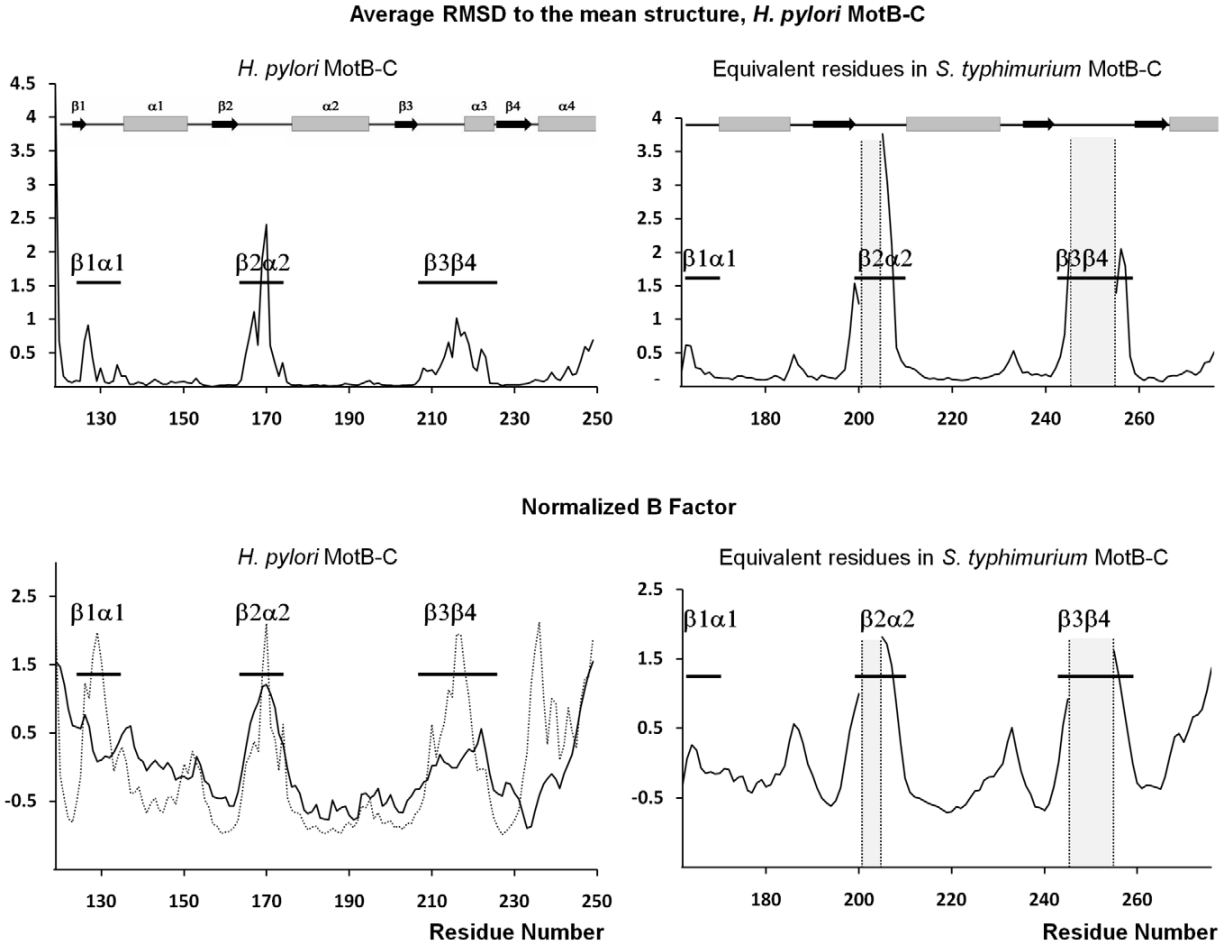
The analysis of the conformational flexibility of MotB-C. A: Average Cα atom RMSD to the mean structure as a function of residue number. RMSD for *H. pylori* MotB-C was calculated with the superimposition of the total of 16 monomers in the asymmetric units of Form A and Form B crystals. RMSD for *S. typhimurium* MotB-C was calculated with the superimposition of the total of five monomers in the asymmetric units of the three available crystal forms (PDB accession codes 2ZOV, 2ZVY and 2ZVZ [7]. Residues 201–204 and 246–254 are disordered in some *S. typhimurium* MotB-C monomers and were therefore excluded from calculations. The positions of the secondary structure elements (α-helices and β-strands) are shown on top. B: Experimental (crystallography, solid line) and theoretical (MD simulations, dotted line) normalized main-chain temperature factor *B. B* equals 8/3π^2^ *u* ^2^, where *u* ^2^ is the mean-square displacement of an atom about its mean position. The crystallographic *B* values for *H. pylori* MotB-C have been averaged over 12 monomers in the asymmetric unit of the Form B crystal. The crystallographic *B* values for *S. typhimurium* MotB-C have been averaged over 5 monomers in the three crystal forms. The *B*-factors were normalized to zero mean and unit variance. The positions of the three carbohydrate-binding loops are indicated.

One well recognized approach to obtain information about conformational movements in proteins is comparison of structures of independent monomers in the crystallographic asymmetric unit or comparative analysis of different crystal forms of the same molecule [14]–[16]. To compare the structures of the individual monomers in the asymmetric units of the Form A and Form B crystals, the monomer structures were superimposed and analysed for Cα atom root-mean-square-deviation (RMSD) to establish regions of conformational variability. The most significant differences are observed in the structures of loops β1α1, β2α2 and β3β4 at the PG binding site (Fig. 1(A), 2(A)), with the following residues showing the largest displacements of Cα atoms: Glu126 (2.3 Å), Asn127 (2.4 Å) (loop β1α1), Val169 (2.8 Å), Lys170 (4.1 Å) (loop β2α2) and Asp216 (2.4 Å) (loop β3β4). Analysis of the distribution of the main-chain temperature factor averaged over the total of 12 monomers in the asymmetric units of the Form B crystals also highlights these three loops as the most variable regions in the structure (Fig. 2(B)). Furthermore, the monomer structure superimpositions and the temperature factor analysis over the equivalent residue range in the three available crystal forms of *S. typhimurium* MotB-C [7] (Fig. 2) identifies the loops equivalent to loops β2α2 and β3β4 in *H. pylori* protein as highly mobile thus supporting the hypothesis that the loop movements at the PG-binding site are a universal feature of MotB proteins.

Structure superimposition shows that there are significant differences in conformations of many side chains and the orientation of the main-chain peptide groups in loops β1α1, β2α2 and β3β4. These differences are linked to the differences in the networks of hydrogen bonds that stabilize the loop structure. For instance, in one of the conformations, the side chain of Asp127 forms a hydrogen bond with the main-chain peptide of Ala128, whereas in a different conformation, it is hydrogen-bonded to the main-chain NH-group of Thr129. Reorientation of the main-chain peptide groups leads to formation/breakage of the hydrogen bonds Ala128(O) - Arg-183(Nε), Leu168(O) - Thr171(N), Leu168(O) - Thr171(Oβ), Asn215(O) - Arg221(Nε), Asp216(O) - Arg221(Nζ) and Asp216(Oγ) - Asn220(Nδ). All the loop residues that show side-chain conformational variability in the analysed crystal structures have been previously implicated in PG binding. Residues 126–129 form a stretch that is structurally equivalent to *H. influenzae* PAL residues Gly35, Phe36 and Asp37, the latter two being involved in binding to the peptide moiety of a PG precursor [9]. Residues 168–171 and 215–216 belong to loops β2α2 and β3β4 which in turn, have been implicated in recognition of the glycan chain of PG [6]. The length and the sequence of the long loops show significantly less cross-species variation than those of the short loops (α1β2, α2β3 and β4α4) at the opposite end of the β-sheet (Fig. S1). Thus, our crystallographic analysis suggests that the flexibility of the semi-conserved loop region β1α1, β2α2 and β3β4 is important for PG-binding activity of MotB.

The biological function of proteins requires the ability to change conformation [17]. In particular, the structural flexibility has been associated with molecular recognition [18]. Flexibility around the ligand-binding site improves its accessibility, facilitates the ligand entry and allows subsequent optimisation of the surface complementarity to maximise the number of contacts between ligand and protein upon binding. The variations of MotB petal-like loops β1α1, β2α2 and β3β4 identified through our crystallographic analysis are thought to reflect natural flexibility at this site required for insertion into the PG mesh. Indeed, it has been previously established that the size of the MotB-C dimer is very close to the size of the pore in the PG mesh (approximately 70 Å) [6], [19]. Therefore, the PG-binding surface on MotB-C must be flexible enough to allow its insertion into the PG pore. This hypothesis can be tested by introducing rigidity into the PG-binding loops (*via* proline substitutions or engineered disulfide bridges) and then testing the activity of the resultant MotB mutants *in vivo*. A useful insight into conformational rearrangements in MotB required for peptidoglycan (PG) binding can also be gained through determination of the structure of MotB-C complex with a peptidoglycan fragment.

### Role of the concerted motions of the petal-like loops in MotB function

An important functional aspect of the loop flexibility at the PG binding site lies in their ability to mask/unmask the five conserved residues critical for PG recognition. In order to understand how concerted motions of these loops may expose the cluster of the buried conserved residues (Fig. 1(B)), we performed mass-weighted principal component analysis (PCA) of the molecular dynamics (MD) simulation of the MotB-C dimer. We chose to perform PCA of MD simulations rather than crystal structures to avoid bias imposed by crystal contacts. For validation, theoretical *B*-factors have been calculated for the MD trajectory and showed a very similar trend to the crystallographic B-values (Fig. 2(B)). PCA produces so called principal modes revealing the concerted motions within a molecule and their directions. Typically, the first few principal modes explain most of the motion observed during the MD simulation [20]. Here we describe the loop motions in two chains comprising the dimer along the first three eigenvectors, which account for 44% and 42% of the overall motion of chains D and E respectively.

**Figure 3:**
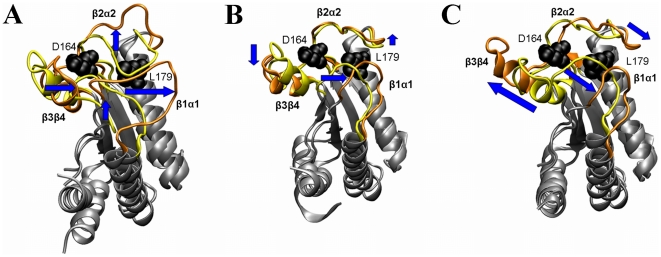
MD motion profiles 1 to 3 along the first three PCA eigenvectors. The two extreme projections of the loops are shown for each motion profile. These extreme conformations (yellow and orange) correspond to artificially heated (2kT above the ground state) conformations for ease of interpretation. The arrows represent the directions of motions between the extreme conformations (here arbitrarily taken as yellow to orange). The side chains of Asp164 and Leu179 are shown in black using a ball representation.

Chain D mode 1 (motion profile 1 (Fig. 3(A))) accounts for 24% of the overall motion of this chain. In this mode, part of loop β1α1 moves in concerted fashion with loop β3β4 and in an opposite direction to loop β2α2 and residues 126–128 in loop β1α1. Upon this movement, the grooves harbouring conserved MotB residues (including Asp164 and Leu179 implicated in binding to the peptide moiety of PG) open up making them accessible to PG. Motion profile 2 (Chain E mode 2, Fig. 3(B)) accounts for 13% of the overall motion and is dominated by the movements of loops β2α2 and β3β4 resulting in the opening of the cleft between these two loops. This could increase accessibility of Asp164, and Leu 179 to a lower extent. The motions of the petal-like loops in modes 2 and 3 from chain D (accounting for 20% of the overall motion) and modes 1 and 3 from chain E (accounting for 29% of the overall motion) show only minor differences in their extent and exact directions and are therefore grouped into a single motion profile 3 (Fig. 3(C)). In this motion profile, loops β2α2 and β1α1 move concomitantly in nearly opposite direction to loop β3β4. This movement is likely to promote cleft widening between β3β4 and β2α2, exposing Asp164.

Thus, the results of the MD simulations are consistent with and also extend the crystallographic analysis, and provide evidence that the three loops move in a concerted fashion, likely to expose conserved MotB residues that have previously been implicated in binding of the peptide moiety of PG. The intrinsic conformational variability of the surface-exposed PG-binding residues and the evidence of intramolecular motions promoting exposure of the buried ones suggest that PG recognition by OmpA-like proteins may occurs *via* a conformational selection rather than an induced fit mechanism.

## Materials and Methods

### Protein production, crystallization and data collection

Recombinant *H. pylori* MotB-C was expressed in *E. coli* and purified as described previously [8]. Protein was concentrated to 8 mg/ml (based on the Bradford assay [21]) and centrifuged for 20 min at 13,000 g to clarify the solution. The crystals have been obtained by the sitting-drop vapour-diffusion method using the drops containing 3 µl of the protein solution mixed with 3 µl of the reservoir solution of 15% PEG 3350 and 200 mM sodium tartrate, and equilibrated against 500 µl of the reservoir solution at 293 K. For data collection, crystals were flash-cooled to 100 K after soaking in a cryoprotectant solution containing 22% PEG 3350, 200 mM sodium tartrate and 20% (*v/v*) glycerol. X-ray diffraction data were collected to 2.5 Å resolution using the Swiss Light Source (PX06, Villigen, Switzerland), and processed and scaled using programs MOSFLM [22] and SCALA [23] (see Table S1). These crystals belong to space group *P*2_1_ with unit-cell parameters a = 107.6, b = 100.3, c = 108.5 Å, β = 119.5°.

### Structure Determination and Analysis

The structure of the new crystal form of MotB-C was solved by molecular replacement using AMORE [10] with a crystallographic P222 tetramer observed in the previously reported form (RCSB PDB code 3CYP [6]), as a search model. The asymmetric unit contains three tetramers related by a pseudo three-fold symmetry about the axis approximately parallel to *b*. Model building and refinement were carried out using programs COOT [24] and REFMAC (CCP4 [23]), without non-crystallographic symmetry restraints. The REFMAC TLS refinement option was employed, with each individual monomer treated as one entity. The final protein model was validated using MolProbity [25]. Final refinement statistics is summarized in Table S1.

Structure superpositions were performed using the program Theseus [11]. Sequence alignment was carried out using the software CLUSTALW2 (http://www.ebi.ac.uk/Tools/clustalw2/index.html). Accessible surface area was calculated using Areaimol in CCP4 [23] with a probe radius of 1.4 Å.

### Molecular dynamics (MD) simulations and PCA analysis

MD simulations were performed on the MotB-C dimer comprising chains D and E of the high-resolution crystal structure PDB 3CYP [6] using NAMD2 (version 2.7b1) [26] and the CHARMM27 force field with CMAP correction [27]. Missing hydrogen atoms were added using VMD [28]. The protein was then solvated into a 86 Å ×86 Å ×86 Å box of water (TIP3 model). This resulted in a chargeless system of 59,822 atoms. After minimisation and equilibration of the water and ions with the protein fixed the system was equilibrated in an NPT ensemble (P = 1.0 bar; T = 310 K) for 1 ns where positional constraints on the protein were smoothly relaxed. The simulation was run for 126.5 ns under the same conditions with periodic boundary conditions and a time step of 2 fs. Van der Waals interaction cutoff was set to 12 Å and long-range electrostatic forces were computed using the particle-mesh Ewald summation method. Mass-weighted PCA (or quasiharmonic analysis) [29] of the MD trajectory was used to determine the principal components of motion for each MotB-C monomer within the dimer (5 ps frame rate). Mass-weighted covariance matrices of each monomer were diagonalised and the principal modes extracted using *ptraj* (AmberTools) [30]. The first three modes were then individually projected onto the Cartesian space.

### Accession codes

Coordinates and structure factors have been deposited to PDB RCSB with the accession code 3IMP.

## Supporting Information

Figure S1 **Alignment of representative sequences for the C-terminal domains of MotBs.** The sequences are shown for *H. pylori* 26695 (Hp; UniProt P56427), *Sulfurimonas denitrificans* (Sd; UniProtKB/TrEMBL Q30RT7), *Borrelia burgdorferi* ZS7 (Bb; SWISS-PROT/TrEMBL Q57371), *Rhodobacter sphaeroides* WS8 (Rs; UniProtKB/TrEMBL A3PKW2), *Pseudomonas putida* GB1 (Pp; NCBI-GI 167034765), *Bacillus subtilis* (Bs; UniProtKB/Swiss-Prot entry P28612), *Aquifex aeolicus* VF5 (Ae; SWISS-PROT/TrEMBL O67121) and *Escherichia coli* (Ec; UniProtKB/Swiss-Prot entry P0AF06). Sequence numbering is shown for *H. pylori* MotB-C. Conserved residues are highlighted in red. In the LOGO representation of alignment above the sequences, the size of the letter denotes a residue's relative conservation among homologues.
(TIF)

Table S1 **X-ray data collection and refinement statistics.**
(DOC)
