# Supplementary figures and images for: Crystallographic and Molecular Dynamics Analysis of Loop Motions Unmasking the Peptidoglycan-Binding Site in Stator Protein MotB of Flagellar Motor
Source: PLoS One. 2011 Apr 20;6(4):e18981. doi: 10.1371/journal.pone.0018981 (PMC3080404; doi:10.1371/journal.pone.0018981)

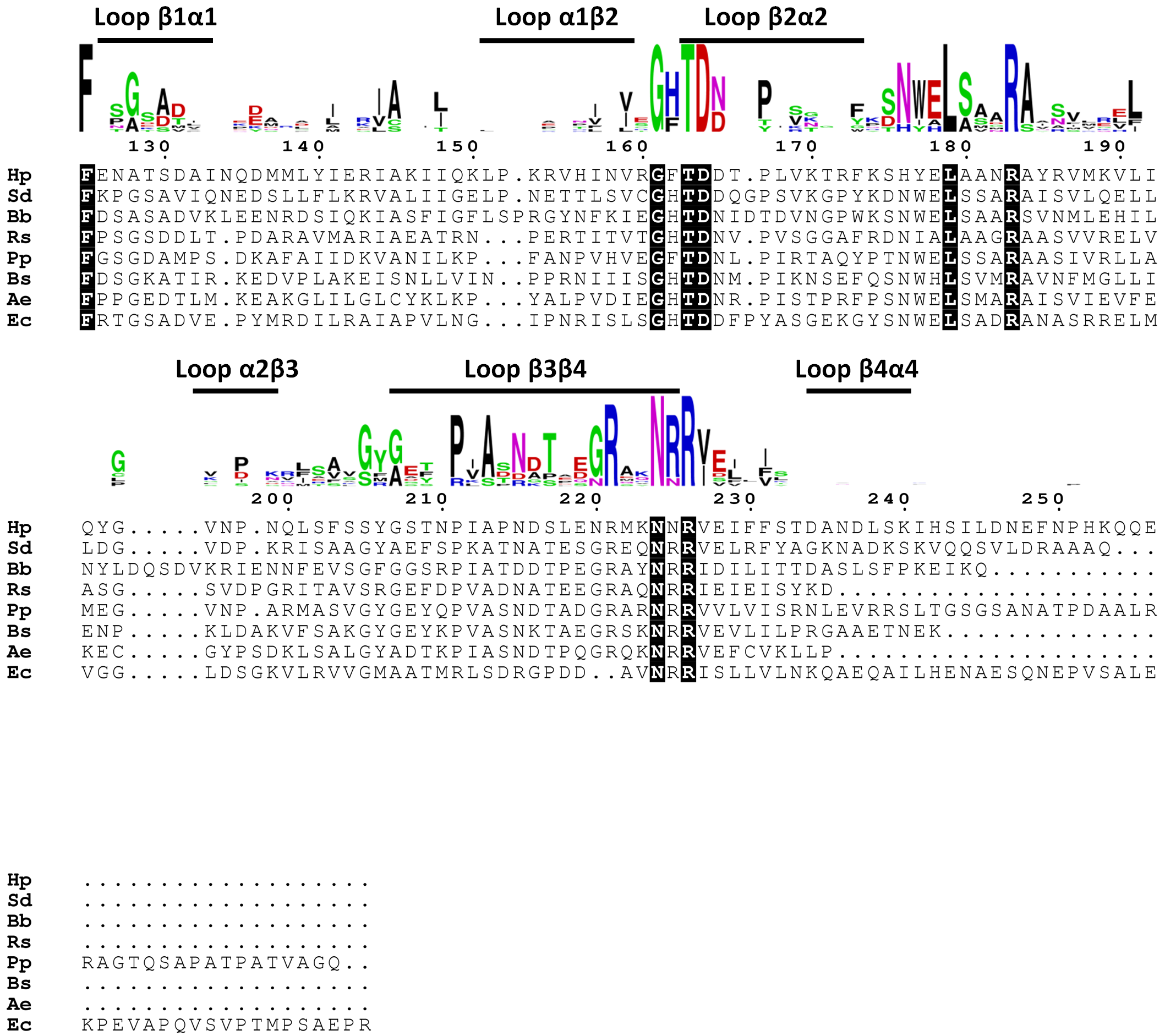

Supplement: Figure S1 — Alignment of representative sequences for the C-terminal domains of MotBs. The sequences are shown for H. pylori 26695 (Hp; UniProt P56427), Sulfurimonas denitrificans (Sd; UniProtKB/TrEMBL Q30RT7), Borrelia burgdorferi ZS7 (Bb; SWISS-PROT/TrEMBL Q57371), Rhodobacter sphaeroides WS8 (Rs; UniProtKB/TrEMBL A3PKW2), Pseudomonas putida GB1 (Pp; NCBI-GI 167034765), Bacillus subtilis (Bs; UniProtKB/Swiss-Prot entry P28612), Aquifex aeolicus VF5 (Ae; SWISS-PROT/TrEMBL O67121) and Escherichia coli (Ec; UniProtKB/Swiss-Prot entry P0AF06). Sequence numbering is shown for H. pylori MotB-C. Conserved residues are highlighted in red. In the LOGO representation of alignment above the sequences, the size of the letter denotes a residue's relative conservation among homologues. (TIF) [file pone.0018981.s001.tif]
