# Supplementary material for: Crystallographic and Molecular Dynamics Analysis of Loop Motions Unmasking the Peptidoglycan-Binding Site in Stator Protein MotB of Flagellar Motor
Source: PLoS One. 2011 Apr 20;6(4):e18981. doi: 10.1371/journal.pone.0018981 (PMC3080404; doi:10.1371/journal.pone.0018981)
Supplement: Table S1 — X-ray data collection and refinement statistics. (DOC) [file pone.0018981.s002.doc]

**Table S1. X-ray data collection and refinement statistics**

| ***Data collection*** |  |
| --- | --- |
| Wavelength (Å) | 0.9 |
| Resolution range (Å) | 15-2.5 (2.64-2.50) |
| Completeness (%) | 99 (99) |
| Observed reflections | 228,866 |
| Unique reflections | 69,194 |
| Average I/σ(I) | 10.4 (2.8) |
| ^a^Rmerge | 0.105 (0.363) |
| ***Refinement*** |  |
| Resolution range (Å) | 15-2.5 (2.64-2.50) |
| Reflections | 65,678 |
| Residues/atoms/waters | 1,586/13,288/433 |
| ^b^R-factor | 0.186 |
| ^c^Free R-factor | 0.249 |
| Average B (protein) (Å^2^) | 43 |
| Average B (water) (Å^2^) | 54 |
| Bond-length deviation from ideality (Å) | 0.014 |
| Bond-angle deviation from ideality (º)  ***^d^Molprobity scores***  Clash score  Clash score percentile  Ramachandran space (%)  Favored  Allowed  Outliers | 1.4  13.5  90  96.4  3.1  0.5 |

^a^, where is the intensity of the ith observation of reflection h.

Numbers in parentheses indicate values for the highest resolution shell.

b

^c^The free R-factor was calculated on 5% of the data omitted at random.

^d^Reference [25].
